# Aspergillus-PCR in bronchoalveolar lavage for detection of invasive pulmonary aspergillosis in immunocompromised patients

**DOI:** 10.1186/1471-2334-12-237

**Published:** 2012-10-02

**Authors:** Michael Buess, Gieri Cathomas, Jörg Halter, Lilian Junker, Peter Grendelmeier, Michael Tamm, Daiana Stolz

**Affiliations:** 1 Clinic of Pulmonary Medicine and Respiratory Cell Research, University Hospital Basel, Basel, Switzerland; 2 Clinic for Hematology, University Hospital Basel, Basel, Switzerland; 3 Department of Microbiology, Kanton Hospital Liestal, Liestal, Switzerland

**Keywords:** Invasive aspergillosis, Lung infection, Diagnosis, Bronchoscopy, Conventional nested PCR

## Abstract

**Background:**

Invasive fungal disease (IFD) is a frequent and serious infectious complication in immunocompromised patients. Culture and cytology in bronchoalveolar lavage (BAL) have a high specificity but low sensitivity for the diagnosis of IFD as assessed by histology. Molecular methods are expected to allow a rapid diagnosis of IFD with a high sensitivity. We evaluated the diagnostic accuracy of conventional nested PCR in the bronchoalveolar fluid to diagnose IFD in severely immunocompromised patients.

**Methods:**

Consecutive immunosuppressed patients undergoing bronchoscopy for suspected pulmonary infection in a tertiary care hospital were included. Patients were classified as having “proven”, “probable”, “possible”, and “no” IFD based on definitions of the European Organization for Research and Treatment of Cancer and National Institute of Allergy and Infectious Diseases (EORTC/NIAID) and on clinical grounds. Conventional nested PCR for aspergillus fumigatus, flavus, niger, glaucus, terreus and tomarrii were applied to 2.5 ml bronchoalveolar fluid.

**Results:**

A total of 191 patients were included. Mean age was 51 y, 61% were male. There were 129 patients with hematological conditions, 26 solid organ transplant recipients, 24 auto-immune disorders, and 12 HIV. According to the EORTC/NIAID classification, there were 53 patients with potential IFD: 3 (2%) had proven, 8 (4%) probable, 42 (22%) possible and 138 (72%) no IFD. A total of 111 (58%) of the patients - 10 (90.9%) proven or probable IFD, 32 (76.2%) possible IFD and 69 (50%) “no” IFD) were on anti-fungal therapy at the time of bronchoscopy. Conventional nested PCR for Aspergillus was positive in 55 cases (28.8%). According to these results, sensitivity, specificity, PPV and NPV for “proven” IFD was 0%, 71%, 0%, 98%, respectively and “probable” IFD was 36%, 72%, 7%, 95%, respectively. In 53 (28%) cases there was a strong clinical suspicion of IFD in the chest-x-ray and/or chest-CT irrespective of the EORTC/NIAID classification. However, from those, only 15 (28%) had a positive conventional nested PCR.

**Conclusion:**

In our experience, conventional nested Aspergillus PCR in the BAL seems to be of limited usefulness for detection of invasive fungal disease in immunocompromised patients due to the limited sensitivity and specificity of the method.

## Background

Invasive fungal disease (IFD) is a frequent and serious infectious complication in immunocompromised patients. Despite the introduction of voriconazole and other new antifungal agents the mortality remains as high as 30% [1]. For instance, the treatment of invasive aspergillosis with intravenous antifungal therapy produced median hospital costs of $52 803 between 2000 and 2006 in the US, although the mortality stayed up to 36.7% [2].

Typical features of IFD on CT scan include the halo sign or cavities. However, in many cases, there are only unspecific infiltrates. Culture and cytology in BAL have a high specificity (70–95%) but low sensitivity (0–88%) for the diagnosis of IFD in neutropenic patients as assessed by histology [3]. Serum galactomannan has been suggested as a non-invasive marker for the diagnosis of IFD in neutropenic patients [4,5]. Conversely, a number of studies showed prominent false positive results [6,7]. The reported sensitivity of serum galactomannan ranges from 50 to 73% [8-10]. Herein, bronchoalveolar lavage galactomannan probably has a higher sensitivity than serum galactomannan [9]. Nevertheless, the specificity of serum galactomannan was 98% or higher in most collectives studied [11].

Currently, the gold-standard for diagnosis of invasive pulmonary aspergillosis remains the direct examination or culture of pulmonary tissue obtained by transbronchial or open lung biopsy [12]. Unfortunately, these procedures might be associated with considerable morbidity and mortality in this particular patient population. While transbronchial biopsies are considered safe in non-immunosuppressed patients with a normal coagulation systems [13,14] decreases in the platelet function or number are linked to a considerable increase in peri-interventional parenchymal bleeding [15]. Surgical biopsy reliably allows detecting pulmonary IFD in a diagnostic and therapeutic approach [16,17]. However, both procedures require specialized, experienced physicians and institutions. Therefore, the development of a reliable, sensitive method to confirm the presence of invasive pulmonary aspergillosis would represent a desirable advance for the adequate treatment of patients at risk [12].

Molecular diagnostic methods for detection of fungal infection have been described in the immunosuppressed population. Khot et al. reported sensitivity, specificity, positive and negative predictive values of 77%, 88%, 50%, 96%, respectively, for a quantitative PCR in the bronchoalveolar lavage (BAL) of 81 patients with hematological malignancies or undergoing hematopoietic cell transplantation [18]. Tuon et al. reviewed 15 studies using PCR in BAL samples to detect invasive aspergillosis and described overall an sensitivity and specificity values of PCR-based techniques to be 79% and 94%, respectivel [19]. Although major concerns related to the lack of external standardization, wide plethora of different methods (real-time, nested, etc.) and need for larger studies have been widely acknowledged, [19] the molecular diagnostic in the BAL seems to be a promising approach for the diagnosis of IFD. Hence, harmonization efforts are ongoing in order to help integrating PCR into the next revision of the EORTC classification [20].

In this study, we aimed to evaluate the diagnostic value of conventional nested PCR to detect aspergillus fumigatus, flavus, niger, glaucus, terreus and tomarrii in the bronchoalveolar fluids in a well-characterized, large population of immunosuppressed patients.

## Methods

This is a prospective, investigator-initiated and –driven, observational, monocentric cohort study. Data of 191 immunocompromised patients undergoing flexible bronchoscopy over a two year period at the Clinic for Pulmonary Medicine, University Hospital Basel, a 784-bed tertiary care hospital located in Basel, Switzerland were analyzed. The primary goal of the study was to evaluate the diagnostic performance of conventional nested PCR to detect aspergillus fumigatus, flavus, niger, glaucus, terreus and tomarrii in the bronchoalveolar fluids for the diagnosis of invasive fungal disease in immunosuppressed patients.

Inclusion criteria were: a) age ≥ 18, b) immunocompromised state as defined by hematologic or other malignancy treated by high-dose chemotherapy and/or stem cell transplantation; solid organ transplantation; AIDS; long-term corticosteroid therapy (i.e., > = 20 mg/d prednisone equivalent for > = 2 months), current use of immunosuppressive, or cytotoxic medication for indications other than organ or stem cell transplantation; c) decision by the pulmonary consultant to perform bronchoscopy due to suspected pulmonary infection; d) informed consent by the patient to undergo flexible bronchoscopy and related data analysis. Exclusion criteria were pregnancy and inability to provide informed consent. The study was approved by the Basel Ethics Committee. All patients gave written informed consent.

### Definitions

Suspected pulmonary infection was defined by the presence of respiratory symptoms, fever and/or new or progressive radiology findings, as judged by the attending physician.

Flexible bronchoscopy was performed with the patient under conscious sedation using hydrocodone and midazolam according to the British Thoracic Society guidelines [21]. BAL was performed by the three installations of 50 mL each of a pyrogen-free, sterile, 0.9% NaCL solution over the working channel of the bronchoscope according to standard guidelines [22]. In patients with no or diffuse pulmonary infiltrates, BAL was performed either in the right middle lobe or the lingula. For patients with focal lung infiltrates, BAL was performed in the pulmonary segment corresponding to the radiologic abnormality. BAL fluid was recovered by suction. Besides BAL, the choice of further sampling techniques used during bronchoscopy, such as endo- or transbronchial biopsies or transbronchial needle aspiration, was at the pulmonologist’s discretion.

The microbiological diagnostic workup included a search for Legionella pneumophila, Chlamydia pneumoniae, Mycoplasma pneumoniae, and respiratory viruses (i.e., herpes simplex virus, cytomegalovirus, respiratory syncytial virus, and adenovirus) by polymerase chain reaction, culture, or immunofluorescence. Appropriate stains and cultures for bacteria, mycobacteria, fungi, and Pneumocystis jiroveci were performed. Positive bacterial cultures were counted as the number of colony-forming units per milliliter, and identification and susceptibility tests were performed according to standard methods. Histologic lung biopsy specimens, recovered by transbronchial biopsy, endobronchial biopsy, video assisted thoracic surgery, and autopsy, were obtained according to the clinical setting. Cytologic analysis of BAL fluid was performed with hematoxylin-eosin staining. Cell differentiation in BAL fluid was reported as absolute numbers and as a percentage of the total cell count [22].

Severe neutropenia was defined as an absolute neutrophil count of < 0.5 x 10 ^9^ cells/L.

Patients were examined, treated, and followed up according to the usual practice of the institution. Particularly, the decision to obtain tissue for histological examination was left at the discretion of the attending physician. Chest radiographs were performed in most cases, and CT scans were obtained, as indicated by the treating physicians, in 143 (74.9%) patients. Clinical diagnoses were determined from physician notes, hospital discharge summaries, laboratory studies, radiologic examinations, and pathologic reports.

Invasive pulmonary aspergillosis was defined by the cytologic or histopathologic demonstration of the presence of Aspergillus or the isolation of Aspergillus in a respiratory specimen in the presence of a compatible clinical and radiographic pattern according to a consensus statement of the Invasive Fungal Infections Cooperative Group, European Organization for Research and Treatment of Cancer, and Mycosis Study Group of the National Institute of Allergy and Infectious Disease [23].

### Aspergillus conventional nested PCR

Conventional nested PCR to detect aspergillus fumigatus, flavus, niger, glaucus, terreus and tomarrii was performed in 2.5 ml leftovers of bronchoalveolar fluid. BAL material was centrifuged and DNA extracted from the pellet using EZ1 DNA Tissue Kit (QIAGEN AG, Hombrechtikon, Switzerland) as described by the manufacturer. The presence of adequate DNA was confirmed by amplifying the human beta-globin gene as previously described [24]. Previously described specific sequences of 5.8 sRNA and 18 sRNA of Aspergillus sp. were chosen as target sequences with slight modification [25]. The following primers were used for the detection of Aspergillus DNA: AspNest1 (5’-TCTTGGTTCCGGCATCGAT-3) and AspNest2 (5’TGACAAAGCCCCATACGCT-3’) for the first round and AspNest3 (5’-GAAGAACGCAGCGAAATGC-3’) and AspNest4 (5’-AACACACAAGCCGTGCTTGA-3’) for the second round, respectively, leading to a final PCR product of 146 bp. The basic amplification reactions were done using a volume of 50 μl containing 100 to 500 ng of DNA, 2.5 ml of each primer (final concentration 3 mM), 25 μl Master Mix Gold (Applied Biosystems, Paris, France) [26]. Amplification and detection were performed on the ABI 9800 Fast Thermal Cycler (Applied Biosystem) using for both primer sets, the same cycling conditions with an initial cycling step at 94°C for 10 minutes followed by 94°C, 55°C and 72°C for 30 seconds each ending at 4°C. Ten microliters of each amplification product were analyzed by electrophoresis in an ethidium bromide-containing 2% agarose gel. In addition, PCR products were extracted from a low melting agarose gel and DNA sequencing was performed to confirm the appropriate DNA sequence. Stringent laboratory conditions and appropriate negative and positive controls were performed in each run.

### Statistical analysis

Differences in dichotomous variables were evaluated using the Chi-square test or Fischer’s exact test, as appropriate. Normally distributed parameters were analyzed using the Student’s *t*-test for equality of means. All other continuously non-normally distributed parameters were evaluated using the non-parametric Mann–Whitney *U* test or Kruskal-Wallis test, as appropriate.

The Statistical Package for Social Sciences (SSPS Inc, version 19 for Windows) program was used. All tests will be two-tailed; a p value of <0.05 was considered significant. Results will be expressed as mean (standard deviation) or median interquartile range unless otherwise stated.

## Results

A total of 191 immunocompromised patients were included in the present study. Patient demographics are shown in Table 1. The mean age of the patient cohort was 50.5 years. The most common causes of immunosuppression were hematological malignancies (129 patients, 68%), particularly acute myeloid leukemia (56 patients, 29%) and malignant lymphoma (20 patients, 11%). Median leukocyte counts and CRP were similar in all patient groups (p = ns). Overall, 60 (31%) of the patients underwent stem cell transplantation. Mean duration of hospitalization before bronchoscopy was 11.1 (±18.6) days. The vast majority of patients required bronchoscopy due to fever and cough. Fifty-five percent presented infiltrates in the chest-x-ray, where up only 28% where considered to be suspected of IFD by the radiologists. A total of 85% of the patients were on antibiotics, 58% on antifungal and 35% on antiviral therapy at the time of bronchoscopy.

**Table 1:** Demographics of 191 immunosuppressed patients undergoing BAL for suspicion of Invasive fungal disease, classified according to the EORTC/NIAID

**Patient characteristics**	**All**	**Possible**	**Probable**	**Proven**	**No**
Patient number (%)	191(100)	42(22)	8(4.2)	3(1.6)	138(72.3)
Age, yr	50.5(19–80)	44(22–80)	49.5(24–60)	42.67(29–70)	52.4(19–79)
Male gender (%)	116(60.7)	28(66.7)	4(50)	3(100)	81(58.7)
Symptoms					
Fever (%)	95(49.7)	29(69)	3(37)	1(33.3)	62(44.9)
Cough (%)	97(50.8)	22(52.4)	4(50)	1(33.3)	70(50.7)
Dyspnea (%)	75(39.3)	15(35.7)	4(50)	0	56(40.6)
Sputum (%)	40(20.9)	10(23.8)	0	0	30(21.7)
Chills (%)	17(8.9)	1(2.4)	2(25)	0	14(10.1)
Chest radiograph/CT scan					
Infiltrates (%)	105(55)	24(57.1)	6(75)	2(66.7)	73(52.9)
Suspicion of fungi lesion (%)	53(27.7)	17(40.5)	4(50)	3(100)	29(21)
Stem cell transplantation					
Allogen (%)	48(25.1)	11(26.2)	4(100)	3(100)	30(21.7)
Autolog (%)	12(6.3)	6(14.3)	0	0	6(4.3)
Immunosuppressive therapy					
Steroids (%)	67(35.1)	13(31)	2(25)	0	52(37.7)
Ciclosporine (%)	48(25.1)	13(31)	4(50)	1(33.3)	30(21.7)
Mycophenolate (%)	28(14.7)	4(9.5)	0	0	24(17.4)
Azathioprine (%)	9(4.7)	2(4.8)	1(12.5)	0	6(5.3)
Sirolimus (%)	10(5.2)	2(4.8)	1(12.5)	0	7(5.1)
Tacrolimus (%)	8(4.2)	0	0	0	8(5.8)
Antimicrobial therapy at bronchoscopy					
Antibacterial (%)	163(85.3)	39(92.9)	8(100)	3(100)	113(81.3)
Antiviral (%)	66(34.6)	13(31)	3(37.5)	2(66.7)	48(34.8)
Antifungal (%)	111(58.1)	32(76.2)	7(87.5)	3(100)	69(50)
Disease					
Hematologic disorders (%)	129(67.5)	33(78.6)	8(100)	3(100)	85(61.6)
Solid organ transplants (%)	26(13.6)	4(9.5)	0	0	22(15.9)
AIDS (%)	12(6.3)	0	0	0	12((8.7)
Autoimmune disorders (%)	24(12.6)	5(11.9)	0	0	19(13.8)
Microbiology					
Aspergillus culture BAL (%)	8(4.2)	2(4.8)	2(25)	1(33.3)	3(2.2)
Bacteriology BAL (%)	28(14.7)	3(7.1)	1(12.5)	0	24(17.4)
Positive nPCR BAL (%)	55(28.8)	10(23.8)	4(50)	0	41(29.7)
Galactamannan in blood, ng/ml	0.226(0.1-3)	0.171(0.1-0.5)	0.488(0.1-1.3)	1.1(0.1-3)	0.178(0.1-1.3)
CRP (mg/L)	76.4(0–410)	84.8(0.9-296)	71.8(2.9-240)	61.6(6.8-116)	74(0–410)
Leukocytes (x10^3^/L)	7.8	4.8	2.0	2.1	9.2
BAL cells (x10^6/^L)	313.8	183.6	159.4	678.3	351.6
macrophages (in%)	69.1	71.0	82.4	60.5	68
lymphocytes (in%)	15.7	15.3	11.8	4.0	16.2
neutrophiles (in%)	16.4	9.4	6.7	35.5	18.5
eosinophiles (in%)	0.9	0.8	0.8	0.0	1

Abbreviations: BAL = Bronchoalveolar lavage.

In addition to bronchoalveolar lavage, 15 (7.9%) transbronchial biopsies, 11 (5.8%) endobronchial biopsies and 3 (1.6%) TBNA from the parenchyma have been performed during bronchoscopy. There were no major complications of the procedure. Cytological analysis of BAL revealed a mean of 313x10^6^ cells, hereby 69% macrophages, 16% neutrophiles, 16% lymphocytes, and 1% eosinophiles. There was no difference in the differential cell counts among patients with proven, probable and possible IFD.

Histological confirmation of the diagnosis was achieved in 35 patients. Eight (4.2%) patients were submitted to open lung biopsy. Out of 37 deaths observed in the study, a total of 24 autopsies were performed. In 2 cases without autopsy, further tissue sections were previously obtained for examination (transbronchial biopsy, open lung biopsy, one case each). Thus, histology was available in 57 (29.8%) cases and in 26 (70.3%) of the deceased patients.

### Diagnostic performance of conventional nested Aspergillus PCR according to the EORTC/NIAID classification

According to the EORTC/NIAID classification, “proven”, “probable”, “possible” and “no” IFD was present in 3 (2%), 8 (4%), 42 (22%) and 138 (72%) of the cases, respectively. Therefore, there were 53 patients with potential IFD. Conventional nested PCR in the BAL was positive in 55 cases (29%). From these, 0/3 (0%) in proven, 4/8(50%) in probable, 10/42(24%) in possible and 41/138(30%) in no IFD. Sensitivity, specificity, positive and negative predictive value of conventional nested PCR according to the radiological and clinical suspicion of IFD is presented in Table 2.

**Table 2:** **Diagnostic performance of conventional nested Aspergillus PCR**^**a**^

	***Sensitivity***	***Specificity***	***PPV***	***NPV***
**As compared to EORTC/NIAID**				
Proven	0%	71%	0%	98%
Proven & Probable	36%	72%	7%	95%
Proven & Probable & Possible	26%	70%	26%	71%
**As compared to clinical judgment**				
Receiving antifungal therapy	30%	73%	60%	43%
Suspicion of IFD on radiologic studies	28%	71%	27%	72%

IFD = invasive fungal disease.

In 53 (28%) cases there was a strong clinical suspicion of IFD in the chest-x-ray and/or chest-CT irrespective of the EORTC/NIAID classification. However, from those, only 15 (28%) had a positive conventional nested PCR.

Conventional nested PCR in the BAL was positive in 36/129 patients with hematologic disorders, 8/26 cases of solid organ transplantation, 2/12 patients with AIDS and 9/24 with autoimmune disorders. Thus, the underlying cause of immunsuppression did not significantly influence PCR results in this study (p = 0.605) There was no difference in PCR positivity among allogen (13/48), autolog (5/12) and no (37/131) stem-cell transplant recipients either (p = 0.589).

A total of 111 (58%) of the patients were on anti-fungal therapy at the time of bronchoscopy. Thereby, anti-fungal therapy was administer to 10/11 (90.9%) of the patients with proven or probable IFD and 32/42 (76.2%) patients with possible IFD, respectively. Results of serum galactomannan were assessed in 105 patients (55%). In 4 cases, galactomannan was considered positive (> 0.5 ng/ml). From those, 2 (50%) had a positive PCR results. Galactomannan blood levels were similar across all EORTC/NIAID groups (p = 0.123).

## Discussion

In this study, we report the diagnostic performance of conventional nested aspergillus PCR in the BAL fluid in a well-characterized cohort of 191 immunosuppressed patients. To our knowledge, this is the largest cohort of IFD cases being prospectively analyzed by aspergillus PCR in the BAL. Remarkably and in contrast to previous data, our results fail to support the clinical applicability of aspergillus PCR in the BAL in the immunosuppressed population, mainly due to the lack of sensitivity and specificity of the method.

Our results are in contrast to some but not all previously published studies. While several authors [27-31] suggested the sensitivity of aspergillus PCR in the BAL to range between 80% and 100%, other authors described much poorer results. For instance, Skladny et al. reports a sensitivity of 43% for proven and 33% for probable IFD using BAL PCR [19,32]. Remarkably, false negative results in proven IFD have been described commonly. Buchheidt et al. detected negative PCR results in the BAL of two out of three patients with positive histological findings for invasive Aspergillosis [33] and Khot et al. reported a negative PCR result from one patient with a positive biopsy [18].

The low sensitivity of the assay in our study might have several reasons. Firstly, we have additionally computed the diagnostic performance of the assay by incorporating the group of “possible” IFD as potentially having IFD. As more cases not “formally” suffering from IFD are included as diseased, there is a decrease in the pre-test-probability, thus further reducing the sensitivity of the test from 36% to 26%, as depicted in Table 2. Secondly, previous studies selected patients by definite radiographic findings or pneumonia, thus improving the pre-test probability of the assay [18,29]. Thirdly, the majority of our patient population (almost 60%) was on chronic anti-fungal therapy at the time of bronchoscopy, which might have decreased the number of organisms present in the pulmonary parenchyma and consequently in the BAL fluid, thus impairing identification by BAL PCR [34,35]. Importantly, data on previous fungal therapy are missing in most previous reports [25,29,30,36]. As compared to older studies, it is plausible that recently introduced, highly potent anti-fungal therapy such as voriconazol and new prophylactic treatment schemata’s reduce fungal load to undetectable amounts in the extra vascular compartments. Fourthly, as only selected aspergillus primers have been applied, detection of other fungal microorganisms was not possible using the current assay. Although most IFD cases are related to aspergillus infection, other fungi such as mucormycosis or rhizophus with a similar or identical clinical presentation would have been missed with the selected primers. Fifthly, it is tempting to hypothesize that fungal microorganisms are mainly present in the intravascular space, thus precluding identification in the alveolar space. And finally, we cannot exclude a technical failure of the methods, although the consistency of the findings and the broad experience of the investigators with molecular techniques make this possibility unlikely [37-40].

In the current study, only 3 patients full field the criteria of “proven” IFD according to the EORTC/NIAID classification. Nevertheless, all presented a false-negative result showing a negative PCR for Aspergillus in BAL. In the first case, the patient had been on antifungal therapy over 6 month at the time of bronchoscopy. Surprisingly, with the exception of the positive histology obtained by video-assisted thoracoscopic surgery, clinical EORTC/NIAID classification was negative due to the absence of neutropenia, fever, graft vs. host disease and current steroid therapy, e.g. “no” IFD. In the second case, the patient was on cyclosporine, had a suspicion of fungal infection due to the presence of a coin lesion on thoracic CT-scan as well as a positive aspergillus culture in the BAL, thus fulfilling the “probable” clinical criteria for IFD. The third patient with a proven biopsy had a new “fungi” suspect infiltrate in the CT scan, but low clinical signs (“possible”) for IFD according to the EORTC/NIAID classification. This exemplifies the low agreement between the EORTC/NIAID classification, histological findings and the clinical presentation in IFD. In all three cases, the suspicion of fungal disease raised by the radiologist lead to the initiation of antifungal medication.

While our results suggest an unexpected low sensitivity of the aspergillus PCR in the BAL, the specificity of the test is in line with previously reported data. Historical controls have shown specificities ranging between 84% and 100% [18,29,30]. This can be explained by the small number of proven invasive aspergillosis.

Our study has a few limitations. The limited number of “proven” and “probable” IFD cases hampers robust inferences on the diagnostic performance of the any test due the low prevalence of the disease (=low pre-test probability). Moreover, the lack of a reliable diagnostic gold-standard for IFD in the absence of histological samples and the heterogeneous clinical presentation of the disease limit the diagnostic certainty in the majority of cases. And finally, despite all efforts, we cannot definitively exclude the possibility of a technical failure in the assay procedure.

## Conclusions

In summary, our results suggest a limited clinical applicability of aspergillus PCR in the BAL for the detection of invasive fungal disease (specifically invasive aspergillosis) in a population of immunosuppressed patients.

## Abbreviations

AIDS: Auto Immune Deficiency Syndrome; BAL: Bronchoalveolar Lavage; CT: Computer Tomography; DNA: Desoxyribonucleinacid; EORTC/NIAID: European Organization for Research and Treatment of Cancer and National Institute of Allergy and Infectious Diseases; IFD: Invasive Fungal Disease; nPCR: Conventional nested Polymerase Chain Reaction.

## Competing interests

The authors declare that they have no competing interests.

## Authors’ contributions

MB, MT, DS conceived and designed the study, revised the patients’ charts, analyzed the data, performed statistical analysis and drafted the manuscript. GC carried out the assays and drafted the manuscript. JH, LJ, PG participated in the design and coordination of the study and helped to draft the manuscript. All authors read and approved the final manuscript.

## Pre-publication history

The pre-publication history for this paper can be accessed here:

http://www.biomedcentral.com/1471-2334/12/237/prepub
